# Cardiac hydatidosis: enigmatic presentation of enigmatic diagnosis: a case report

**DOI:** 10.1093/ehjcr/ytag740

**Published:** 2026-10-13

**Authors:** Anurodh Dadarwal, Aditya Kapoor, Shantanu Pande, Ankit Sahu

**Affiliations:** Department of Cardiology, SGPGIMS, Raebreli Road, Lucknow, UP 226014, India; Department of Cardiology, SGPGIMS, Raebreli Road, Lucknow, UP 226014, India; Department of Cardiovascular Thoracic Surgery, SGPGIMS, Raebreli Road, Lucknow, UP 226014, India; Department of Cardiology, SGPGIMS, Raebreli Road, Lucknow, UP 226014, India

**Keywords:** Cardiac hydatidosis, Case report, Surgical excision of cardiac hydatidosis, Pulmonary embolism, Cystic mass, Hydatid cyst

## Abstract

**Background:**

In endemic countries, hydatid disease is often overlooked because of its non-specific clinical presentation and the limited use of multimodality imaging. Unusual anatomical locations and atypical presentations further complicate diagnosis. Multimodality imaging not only helps to confirm the diagnosis but also plays a crucial role in treatment planning.

**Case summary:**

A 45-year-old man from a rural area presented with a 2-year history of haemoptysis and exertional dyspnoea. He had received antitubercular therapy for 1 year without improvement. Subsequent computed tomography angiography at a secondary care centre suggested pulmonary thromboembolism, and he was referred to our centre for further evaluation. He also had a history of foul-smelling cough and peripheral eosinophilia, without any identifiable risk factors for pulmonary thromboembolism. Routine echocardiography revealed a large multi-cystic mass attached to the basal interventricular septum and protruding into the right ventricular outflow tract. Hydatid disease was strongly suspected and subsequently confirmed by serological testing and transoesophageal echocardiography. Cardiac computed tomography and magnetic resonance imaging were performed to better define the nature, location, and extent of the lesion. The patient was treated with albendazole followed by uneventful surgical excision and remains asymptomatic on follow-up.

**Conclusion:**

This case illustrates a rare presentation of cardiac hydatidosis masquerading as pulmonary embolism. Multimodality imaging was essential in characterizing the lesion, defining its anatomic extent, and guiding management. Surgical excision can be performed safely when carefully planned, and perioperative chemotherapy reduces the risk of recurrence.

Learning points

- Cardiac hydatid disease should be suspected in patients from endemic regions when clinical findings or imaging-based clues are present.
- Multimodality imaging (echocardiogram, computed tomography, and magnetic resonance imaging) is crucial for diagnosis and anatomic delineation.
- Surgical excision is the definitive treatment for cardiac hydatidosis and must be planned meticulously to avoid intraoperative complications and recurrence.

## Background

A high index of suspicion is required to diagnose cardiac hydatidosis. In endemic countries, hydatidosis is often undiagnosed because symptoms are non-specific and multimodality imaging is used infrequently. Pulmonary embolism secondary to cardiac hydatidosis is a rare presentation. Multimodality imaging not only confirms the diagnosis but also defines anatomy crucial for definitive management.

## Summary figure

**Figure:**
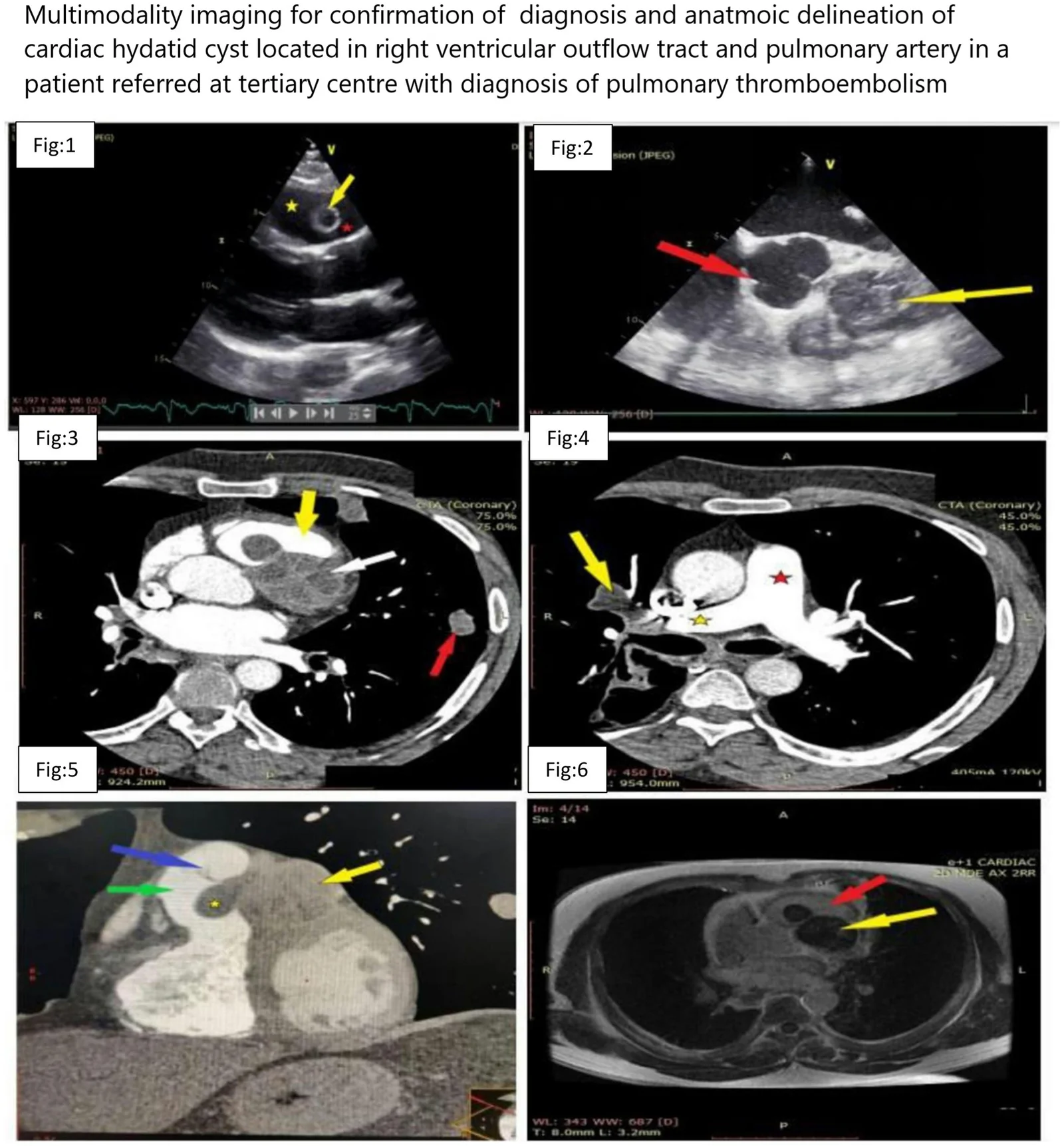


## Case presentation

A 45-year-old male presented to the outpatient department with a 2-year history of haemoptysis, exertional dyspnoea for 18 months, and occasional foul-smelling sputum for 3 months. He denied fever, weight loss, night sweats, orthopnoea, peripheral oedema, bleeding tendency, or productive cough. He had previously received Category I antitubercular therapy for 6 months without symptomatic improvement. The patient had no history of diabetes, hypertension, coronary artery disease, or substance use. Physical examination was unremarkable.

A chest computed tomography (CT) performed at a secondary-care centre reported a mass lesion in the pulmonary artery interpreted as thrombus, and the patient was referred as a case of pulmonary thromboembolism. Laboratory tests were within normal limits except for marked eosinophilia (33%; total leukocyte count 6900/mm^3^). Chest radiography showed multiple ill-defined nodular opacities in the basal lung fields (*Figure 1*). Electrocardiogram (ECG) was normal.

**Figure 1:**
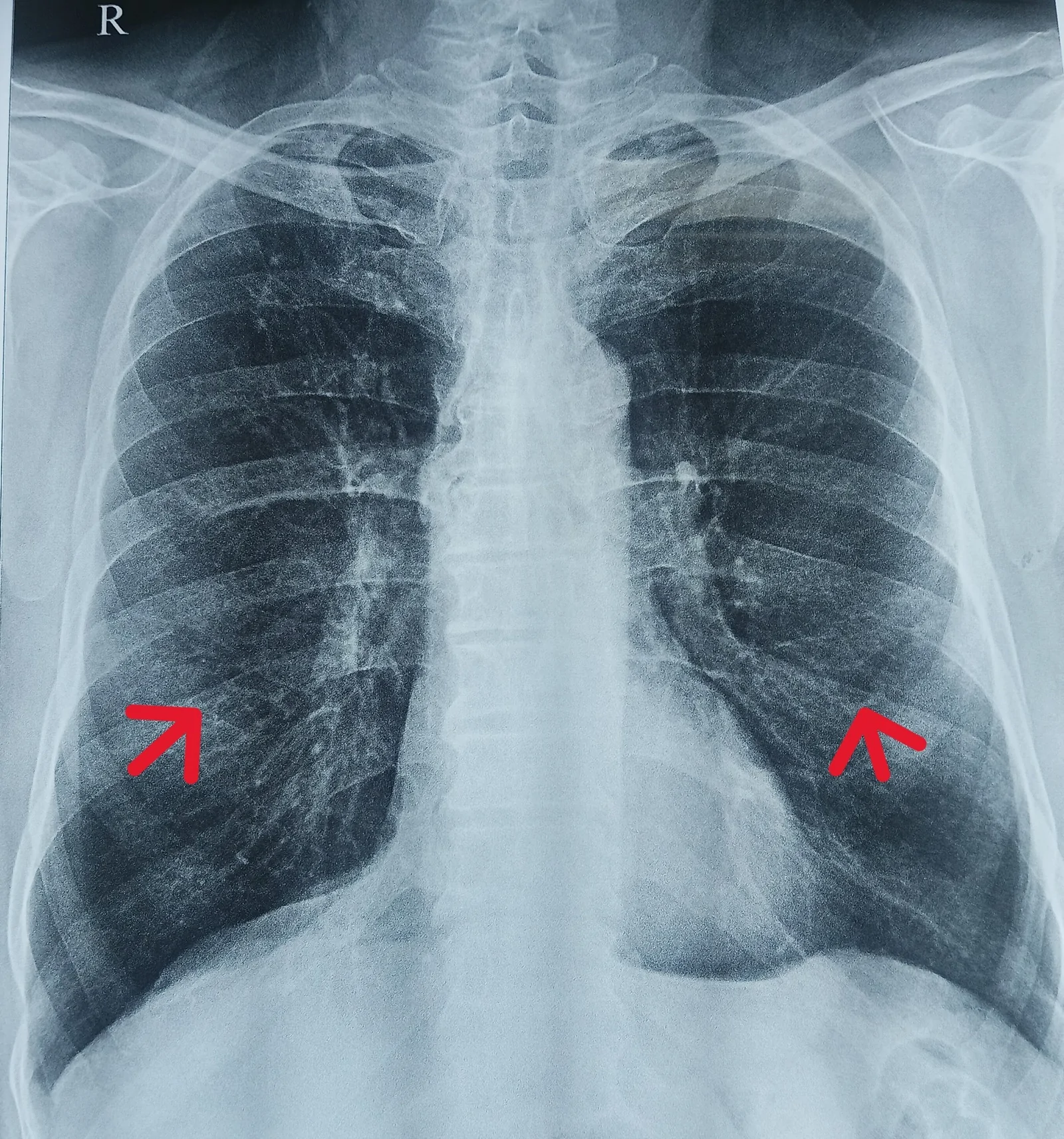
Chest x-ray PA view showing ill-defined cystic mass (red arrow) in left and right lower lung zones.

Transthoracic echocardiography (TTE) revealed a large cystic mass in the right ventricular outflow tract (RVOT) (see Supplementary material online, *Figure S1*, Supplementary material online, *Video S1*) with internal septations (see Supplementary material online, *Figure S2*, Supplementary material online, *Video S2*). Transoesophageal echocardiography (TEE) demonstrated a well-circumscribed cystic lesion in the RVOT immediately below the pulmonary valve (*Figure 2A*, Supplementary material online, *Video S3*).

**Figure 2:**
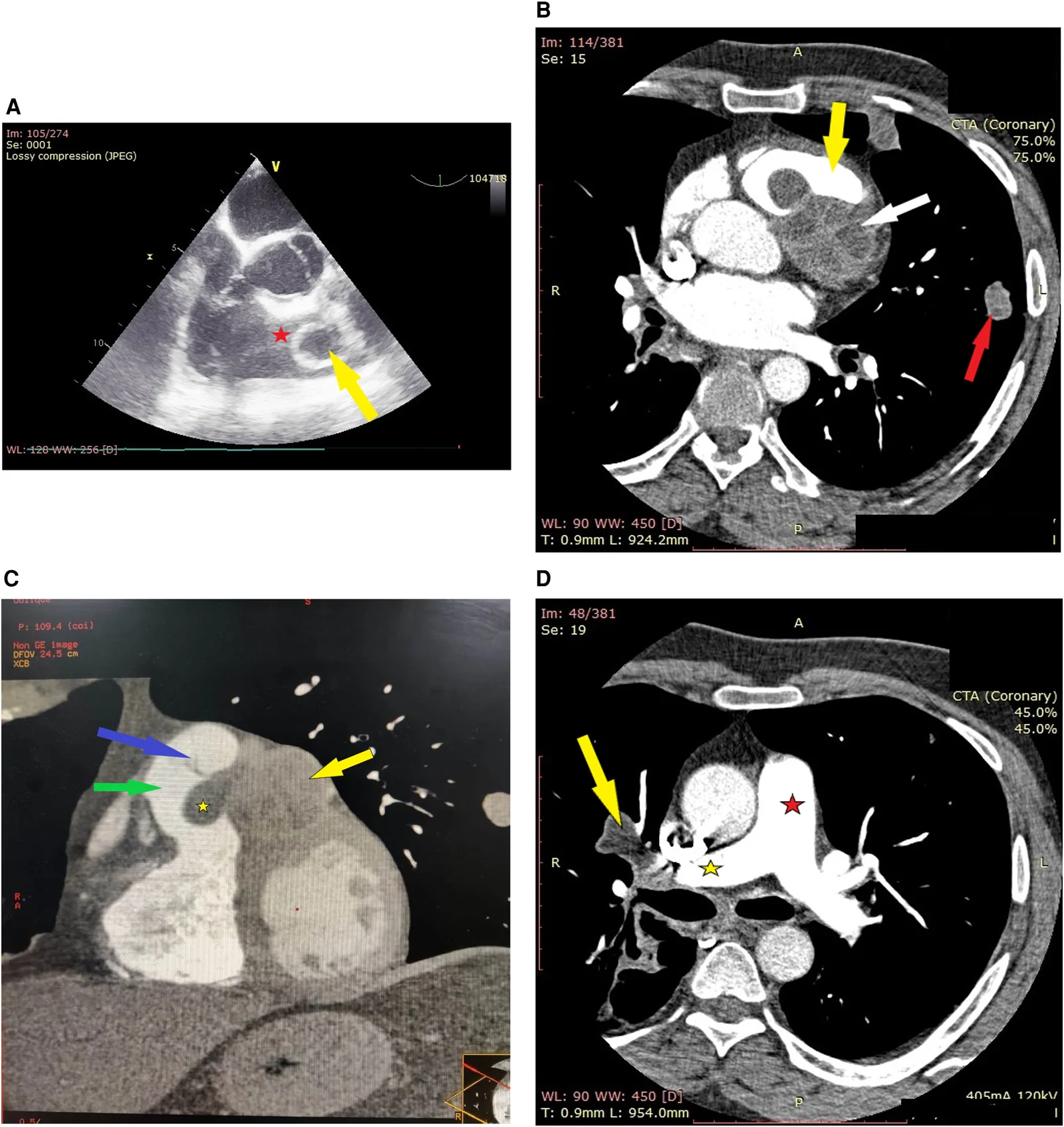
(*A*) Transoesophageal echocardiography right ventricular inflow outflow view: cystic mass (yellow arrow) in right ventricular outflow tract (red star). (*B*) Contrast cardiac computed tomography: axial section showing multi-cystic mass (white arrow, 6.2 × 4.2 × 4.6 cm) in basal septum compressing right ventricular outflow tract (yellow arrow) with similar density multi-cystic lesion (red arrow) in left lung parenchyma. (*C*) Contrast cardiac computed tomography in sagittal section: Multi-cystic lesion (yellow arrow) in basal septum extending (yellow star) in right ventricular outflow tract (green arrow) below pulmonary valve (blue arrow). Note the proximity of the hydatid cyst to the pulmonary valve. (*D*) Contrast –computed tomography—chest: non-enhancing multi-cystic lesion (yellow arrow) in a branch of the right pulmonary artery (note peripheral calcification of the lesion). Right pulmonary artery and main pulmonary artery are marked by a yellow star and a red star, respectively.

Given the history of foul-smelling sputum, haemoptysis, marked eosinophilia, and the cystic echocardiographic appearance, hydatid disease was strongly suspected. Contrast-enhanced cardiac and chest CT was obtained to further evaluate the pulmonary nodules and to exclude thromboembolism. CT (axial scan) of the chest showed a multi-loculated cystic lesion compressing the RVOT and a similar density lesion in the left lung parenchyma (*Figure 2B*). Sagittal images demonstrated a multi-loculated mass attached to the basal septum and protruding into the RVOT just below the pulmonary valve (*Figure 2C*). CT pulmonary angiography revealed a hypodense filling defect with peripheral calcification—attenuation similar to the cardiac lesion—in the anterior and posterior branches of the right pulmonary artery (*Figure 2D*). The mid-interventricular septum was free of cystic involvement (see Supplementary material online, *Figure S3*).

Cardiac magnetic resonance imaging (MRI) further characterized the lesion as a multi-cystic, well-circumscribed, T2-hypointense mass inseparable from the basal septum and protruding into the RVOT (*Figure 3*).

**Figure 3:**
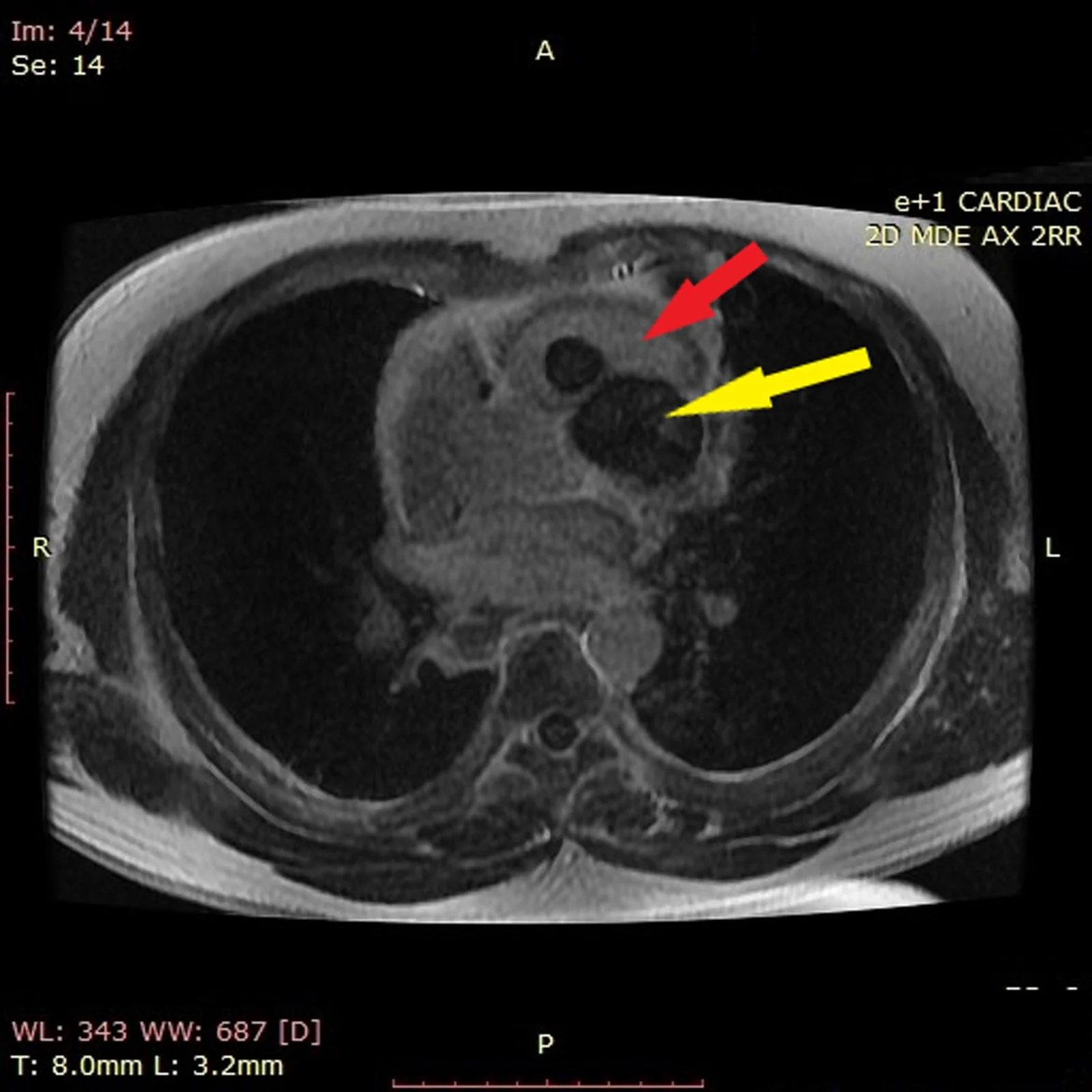
Cardiac magnetic resonance imaging transverse section: hypointense well-defined cystic mass (yellow arrow) in right ventricular outflow tract (red arrow) attached to the basal septum without any extension to the pericardium or adjoining structures.

## Differential diagnosis

Differential diagnosis includes cardiac lipoma, fibroma, myxoma, pericardial cyst, intracardiac thrombus, and blood cyst, and their key differentiating features are given in *Table 1*.^1–5^ Cardiac lipomas are typically subepicardial and appear as homogeneously hyperechoic masses on echocardiography; on CT, they exhibit homogeneous low attenuation, and on MRI ,they are T1 hyperintense with signal loss on fat-saturated sequences and do not enhance with contrast.^1^ Cardiac myxomas involve the ventricle in only 5% of cases, appearing isointense on T1 and hyperintense on T2, with heterogeneous post-contrast enhancement.^1^ Cardiac fibromas are usually seen in paediatric patients and are isointense on T1 and hypointense on T2.^1^ Thrombi appear hyperintense on T1 and T2 in the acute stage and become hypointense in the chronic stage.^1^ Pericardial cysts are commonly located at the right cardiophrenic angle and show well-demarcated homogeneous low or isointense signal on T1 and very high signal on T2-weighted images.^1^ Blood cysts are mostly at valvular locations (mitral valve most commonly), anechoic cystic structure on echo, hypodense on CT, hypointense on T1, and hypertense on T2 sequence in MRI.

**Table 1:** Differential diagnosis and their differentiating features on multimodality imaging

S. No	Disease	Age	Location	Echo	CT	MRI	Contrast enhancement
1	Lipoma	Middle age and older age	LA (subepicardial)	Homogeneous hyperechoic mass	Encapsulated, Homogenous, smooth fat attenuation	T1 hyperintense	Not present
2	Fibroma	Pediatric	LV (septum or free wall) >RV	Hyperechoic	Homogenous low attenuation, calcification frequently present	Isointense on T1 and hypointense on T2	Uniform delayed enhancement
3	Myxoma	40–60 (more in women)	Left atrium > RA > RV > LV	Hyperechoic pedunculated mobile irregular mass attached to fossa ovalis in LA	Pedunculated, mobile, heterogeneous, low attenuation, punctate calcification may be present	Isointense on T1 and hyperintense on T2	Present
4	Pericardial cyst	Congenital, incidentally detected at any age	Right cardio phrenic angle > left cardio phrenic angle	Smooth cystic structure without internal septation	Round or elliptical thin-walled homogenous mass with sharp demarcation and no septation with same density as water	hypointense on T1 weighted and hyperintense on T2 weighted	Not present
5	Thrombus	Variable depend upon cause of thrombus	LAA, LV aneurysm, RA, RV (from DVT embolism)	Homogeneous echo dense and deformable (gel like), old thrombi echo bright and compact immobile	Crescent-shaped filling defect with broad base attachment, low attenuation than contrast enhanced blood pool	Hyperintense on T1 and T2 (acute phase) hypointense in chronic stage)	Not present
6	Blood cyst	variable	In proximity to valve	Well demarcated Anechoic mobile cystic structure	Iso-dense or low-density cystic mass	Iso or hypointense on T1 and hyperintense on T2	Not present

Abbreviations: DVT, deep vein thrombosis; LA, left atrium; LAA, left atrial appendage; LV, left ventricle; RA, right atrium; RV, right ventricle.

Serology with ELISA for *Echinococcus* immunoglobulin G antibodies was positive. Based on clinical history, imaging findings, and positive serology, a diagnosis of cardiac and pulmonary hydatidosis was made.

Because the lesion had a high burden of daughter cysts and was located in the basal septum protruding into the RVOT—features associated with increased intraoperative risk—the patient received oral albendazole 400 mg twice daily (approximately 10–15 mg/kg/day) for 3 months prior to surgery. He subsequently underwent surgical excision under cardiopulmonary bypass without intraoperative or postoperative complications.

Operative findings included a large cyst in the RVOT. The cyst was carefully incised after assessment of surface anatomy and extension through a pulmonary artery window (*Figure 4A*, Supplementary material online, *Video S4*, *Video S5*) and was meticulously drained to avoid spillage, with betadine irrigation, and excised *en bloc* (*Figure 4B*, *Figure 4C*, Supplementary material online, *Video S6*). Marsupialization of the pseudocyst was performed to prevent postoperative refilling with blood or serum and to reduce the risk of RVOT obstruction on follow-up (see Supplementary material online, *Video S7*).

**Figure 4:**
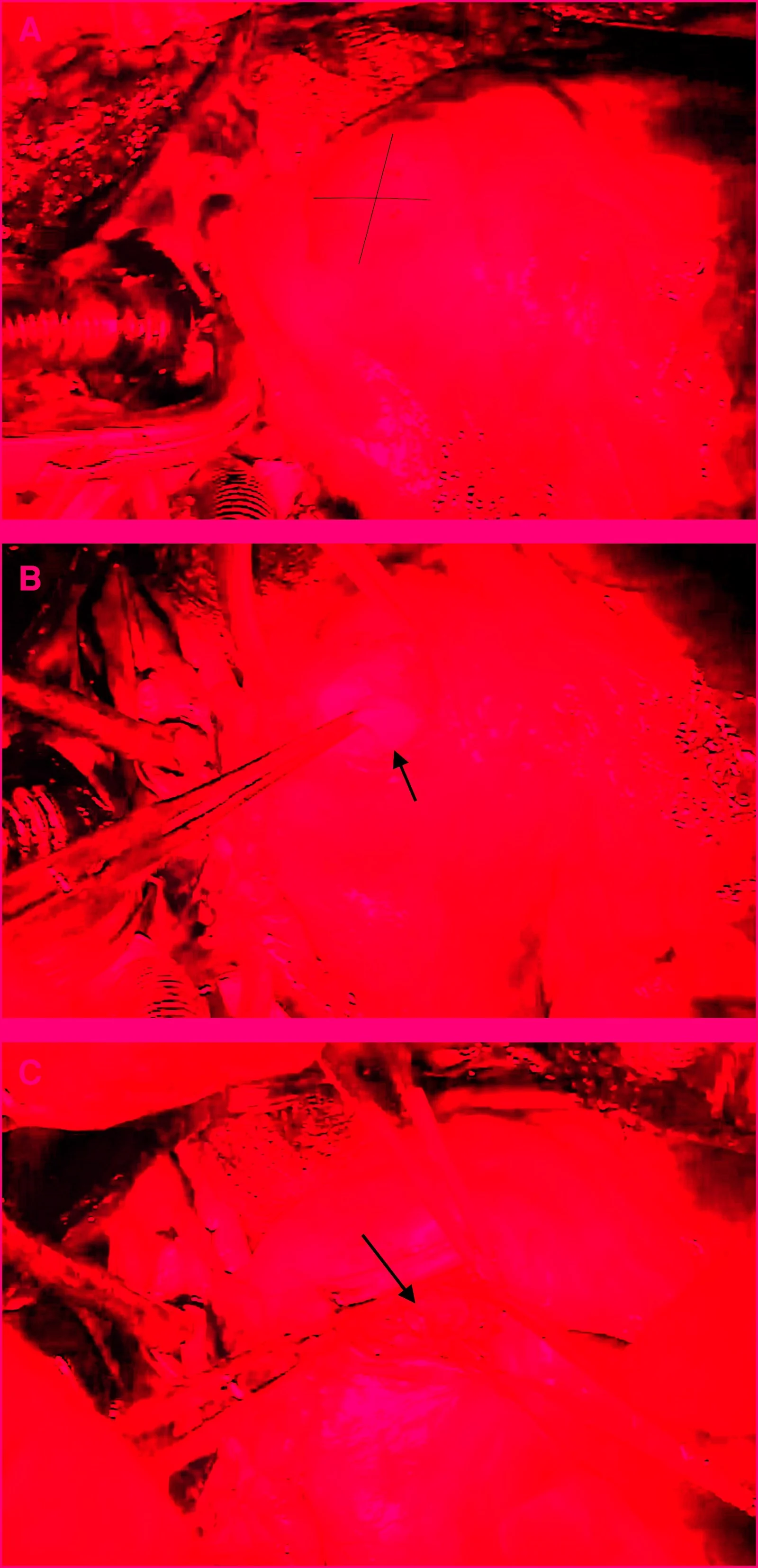
(*A*) Intraop image (after midsternotomy and cardiopulmonary bypass): the extent of the cyst is seen (black cross) from the surface over the right ventricular outflow tract. (*B*) Intraop image (after mid sternotomy and cardiopulmonary bypass): specimen of the daughter cyst being removed from the cyst (highlighted by arrow). (*C*) Intraop image (after mid sternotomy and cardiopulmonary bypass): multiple daughter cysts inside the sac. It is coloured by the use of betadine during operation to kill the cysts.

Postoperative echocardiography showed normal biventricular function without residual cyst or mass. The patient was discharged on postoperative Day 3 on albendazole 400 mg twice daily. At 15- and 45-day follow-up visits, he remained asymptomatic and was doing well.

## Discussion

The World Health Organization estimates the incidence of cardiac echinococcosis at approximately 0.03%–1.1% of all echinococcosis cases.^6^ Approximately 50% of cardiac cases have multi-organ involvement.^7^ Humans acquire infection through close contact with infected carnivores (e.g. dogs) or accidental ingestion of *Echinococcus* eggs. Eggs hatch into oncospheres that can reach the heart via coronary circulation, pulmonary veins, intestinal lymphatics and thoracic duct, superior and inferior vena cava, or even haemorrhoidal veins.^8,9^

Cardiac localization occurs most commonly in the left ventricle (60%), followed by the right ventricle (10%), pericardium (7%), pulmonary artery (6%), left atrial appendage (6%), and interventricular septum (4%).^10^ Pulmonary artery embolism due to hydatid disease is extremely rare and usually results from cardiovascular invasion; it may also occur secondary to invasion of the inferior vena cava or hepatic veins.^11^ Intracavitary cyst rupture is more frequent from the right ventricle than the left and can cause pulmonary embolization, pulmonary hypertension, and death.^12^

Although the disease is asymptomatic in up to 90% of cases, it can present with heart failure, cardiac tamponade, pulmonary embolism, stroke, atrioventricular block, supraventricular tachycardia, mitral regurgitation, pericardial effusion, coronary ischaemia, anaphylaxis, or death. The presence of daughter cysts within a parent cyst is a pathognomonic imaging feature. Diagnosis relies on imaging and serology. TTE and TEE, which demonstrate a cyst with echo-free contents and smooth contours, are efficient screening tools.^13^ CT and cardiovascular magnetic resonance are essential for precise localization and anatomical delineation; CT is useful for detecting calcifications, while MRI offers superior soft-tissue characterization and staging. Serological antibody assays support the radiologic diagnosis.

In this case, the patient, from a rural area and working in grassland, likely acquired infection through close contact with dogs. A high index of suspicion is necessary for similar presentations. Surgical treatment is recommended for cardiac hydatid disease—even in asymptomatic patients—because cyst rupture may precipitate anaphylaxis or metastatic spread.^14^ Antiparasitic therapy with benzimidazoles is an important adjunct to reduce recurrence risk.^15^

## Lead author biography


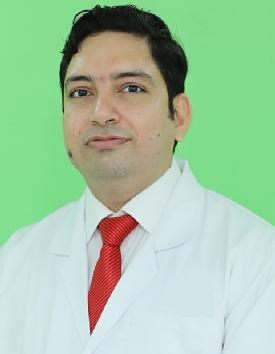


Dr. Anurodh Dadarwal is senior interventional Cardiologist and Director, Cath Lab, Department of Cardiology at Prince Medical College and Hospital, Sikar, Rajasthan, India. He worked as Senior Resident in the Department of Cardiology at SGPGIMS from 2017 to 2021, where the original work for this manuscript was conducted. His clinical interests focus on complex coronary interventions, multimodality imaging and teaching the fellows.

## Supplementary Material

ytag740_Supplementary_Data

## Data Availability

No new data were generated or analysed in support of this case report.
