# Supplementary material for: Cardiac hydatidosis: enigmatic presentation of enigmatic diagnosis: a case report
Source: Eur Heart J Case Rep. 2026 Oct 13;10(10):ytag740. doi: 10.1093/ehjcr/ytag740 (PMC13711670; doi:10.1093/ehjcr/ytag740)
Supplement: Supplementary file 1 [file ytag740_supplementary_data.zip › Supplementary_Legends.docx]

**Supplemental figure 1:** Transthoracic ECHO parasternal long axis view: cystic lesion (yellow arrow) is seen in RVOT (marked by red star) in close proximity to pulmonary valve. Yellow star is denoting RV cavity.

Alternative text: Transthoracic ECHO parasternal long axis view showing cystic lesion in right ventricular outflow tract.

**Supplemental figure 2:** Transthoracic ECHO: During sweep from parasternal long axis view to short axis view multicystic nature of mass (Yellow arrow) is clearly seen in RVOT (red star).

Alternative text: Transthoracic echo showing multicystic nature of mass

**Supplemental Figure 3:** Contrast cardiac CT: cross sectional view showing IVS and left and right ventricle. At this level IVS (yellow arrow), right and left ventricle is free of cyst. Note the heterogenous nodular lesion (red arrow) in right lung.

Alternative text: Cardiac CT further defining the anatomic extension: Hydatid cyst is not involving the whole interventricular septum.

**Video 1:** Transthoracic ECHO parasternal long axis view: cystic lesion is seen in RVOT in close proximity to pulmonary valve.

Alternative text: Transthoracic ECHO showing cystic lesion in RVOT in parasternal long axis view

**Video 2:** Transthoracic ECHO parasternal long axis view: cystic lesion is seen in RVOT in close proximity to pulmonary valve (note the multicystic nature)

Alternative text: Transthoracic ECHO showing multicystic nature of mass in RVOT in parasternal long axis view

**Video 3:** Transesophageal echo with RV inflow outflow view: The cystic lesion in close proximity to pulmonary valve

Alternative text: Transesophageal ECHO showing cystic lesion in RVOT in RV inflow-outflow view

**Video 4:** Intraop Surface marking: after aortic and venous cannulation, surface marking of cyst was done in RVOT and its close proximity to LAD was noted before incision

Alternative text: Intraop surface marking of cyst (in RVOT with close proximity to LAD)

**Video 5:** Inspection of RVOT: The assessment of extension of cyst in RVOT was carefully done through pulmonary artery window

Alternative text: Intraop inspection of cyst in RVOT through pulmonary artery window

**Video 6:** Removal of cyst: Daughter cyst was removed maintaining sterility with betadine wash

Alternative text: Intraop meticulous removal of daughter cysts with betadine wash

**Video 7:** Marsupialization: after removal of all the daughter cysts and contents of hydatid cyst, the pseudocyst was everted and marsupialization was done with continuous sutures to avoid its filling with blood and serum and subsequently possibility of RVOT obstruction.

Alternative text: Marsupialization of pseudocyst after removal of all daughter cysts to avoid of subsequent filling of blood or serum
